# Grandparenting and coresidence preferences in Shanghai, China: the influence of grandparenting beliefs, experiences, and intensity

**DOI:** 10.1093/geronb/gbaf138

**Published:** 2025-07-25

**Authors:** Ying Ma, Qiushi Feng, Zhihong Zhen, Litao Zhao

**Affiliations:** International Development Cooperation Academy, Shanghai University of International Business and Economics, Shanghai, China; Department of Academic Journals, Shanghai University of International Business and Economics, Shanghai, China; Department of Sociology and Anthropology, National University of Singapore, Singapore, Singapore; Department of Sociology, Shanghai University, Shanghai, China; East Asian Institute, National University of Singapore, Singapore, Singapore

**Keywords:** Coresidence preference, Filial norms, Grandchild care, Intergenerational solidarity, Living arrangement

## Abstract

**Objectives:**

This study aims to examine the living arrangement preferences of older adults in Shanghai, China, with a focus on grandparenting as a shaping factor.

**Methods:**

This study utilizes data from the 2022 Lifelong Education for Aging Productively in Shanghai survey, which included 1,707 older adults aged 55–85. Since this study focuses on grandparenting, respondents without grandchildren were excluded, resulting in an analytical sample of 1,250 individuals. In this study, grandparenting is measured through grandparenting beliefs, frequency of grandchild care, and previous grandparenting experiences.

**Results:**

The results indicate a decisive shift toward independent living, with 90% preferring to live alone or with a spouse. However, preferences adapt when adult children require support, including grandparenting. Our analysis shows that grandparenting beliefs, experiences, and intensity are positively associated with the preference for coresidence.

**Discussion:**

These findings highlight the malleability of living arrangement preferences and suggest a persistent, though evolving, form of intergenerational solidarity. This study contributes to the literature by emphasizing the importance of adult children’s needs in shaping parents’ living arrangement preferences in contemporary urban China.

The living arrangements of older adults are a topic of considerable significance, particularly in rapidly modernizing societies such as China. Existing literature highlights the distinction between preferred and actual living arrangements, noting that alignment or misalignment between the two has profound implications for older adults’ well-being (Chen, 2019; Sereny & Gu, 2011; Sun & Zimmer, 2022). Research further suggests that families often adapt living arrangements to meet the evolving needs of aging parents (Chen, 2005; Gu et al., 2019; Zhang et al., 2014; Zimmer, 2005). Yet, less is known about how living arrangement preferences among older adults shift within rapidly modernizing societies where filial norms remain resilient. Even less understood is how these preferences evolve in response to the varying needs of their grown children.

This study aims to address these gaps by examining the living arrangement preferences of older adults in Shanghai, China, with a focus on how the needs of their children, particularly the need for grandparenting, shape their preference for coresidence. Grandparenting has important cultural significance for intergenerational relations in China, which is well aligned with the traditional principle of filial piety in Confucianism and has become highly prevalent when China is modernizing (Chen et al., 2011; Yan, 2018). In the Chinese cultural context, although parent–child relationships are often bound by strict disciplinary expectations and performance pressures, the bond between grandparents and grandchildren is typically characterized by warmth, emotional generosity, and a notable degree of freedom. Recent studies highlight that the duality of grandparenting—rooted in the sense of obligation, yet deeply infused with affectual intimacy—plays a pivotal role in supporting the psychological well-being of both grandparents and grandchildren (Liu, 2017; Xu et al., 2014). With the understanding that grandparenting is multidimensional, this study specifically focused on the instrumental care of the grandchildren.

Shanghai is a particularly illustrative setting for exploring these shifts in living arrangement preferences due to its accelerated aging population, high degree of urbanization, and distinctive cultural and economic context. Designated as China’s first “aging” city, Shanghai’s population over the age of 60 surpassed 10% in 1979, more than two decades before China reached a similar demographic milestone (Huang et al., 2019). Data from the Shanghai Municipal Committee on Aging reveal that by 2023, Shanghai’s aging rate had surged to 37.4%. Notably, the “pure older households,” defined as households where all members are aged 60 or older, comprise over 30% of the older population. The city’s unique blend of traditional Chinese familism and modern individualism, often referred to as “Shanghai culture,” presents a rich landscape for examining older adults’ navigation between the values of family interdependence and personal independence. Insights derived from Shanghai’s older population could potentially forecast living arrangement trends that might extend to other rapidly urbanizing areas in China.

## Literature review

### Actual versus preferred living arrangements

Most research on living arrangements focuses on actual residence patterns. However, there is growing recognition that actual residence is an imperfect proxy for preferred residence. Older adults often face constraints that lead to compromises and mismatches between what they prefer and where they actually live. Such discordance has been shown to negatively impact older adults’ self-rated health, subjective well-being, and overall quality of life (Sereny, 2011; Sereny & Gu, 2011; Sun & ­Zimmer, 2022).

Alongside resources and needs, various formulations have recognized preferences as a key factor shaping intergenerational living arrangements (Fingerman et al., 2020; Gu et al., 2019; Isengard & Szydlik, 2012; Takagi & Silverstein, 2006). Isengard and Szydlik (2012) offer a useful analytical framework that categorizes these factors into opportunity structures (resources available for intergenerational coresidence), need structures (the necessity for help and support through shared living), and cultural-contextual structures (norms and macro-level factors shaping preferences for coresidential living versus independent living).

Although the role of cultural-contextual structures in shaping living arrangements is widely acknowledged, much of the literature assumes a dichotomy: some cultures value independent living, while others prioritize intergenerational coresidence. In Western societies, rising individualism and a preference for autonomy, privacy, and comfort are often cited as reasons for declining coresidence (Lesthaeghe, 1983). Conversely, the persistence of traditional family structures and intergenerational interdependence is seen as central in many developing countries (Bongaarts & Zimmer, 2002; Ruggles & Heggeness, 2008).

This dichotomous view, however, is becoming less tenable. Profound societal changes are reshaping living arrangements even in cultures where independent living has long been the norm. The extended transition to adulthood, alongside challenges such as marital breakdown, unemployment, and housing issues, has increased the prevalence of coresidence (Lamidi & Nash, 2022; Tosi & Grundy, 2018). In East Asia, where filial piety is deeply ingrained, coresidence with adult children is often seen as a filial duty. However, a decline in intergenerational coresidence has been well documented (Takagi & ­Silverstein, 2006; Yasuda et al., 2011).

Recent studies further challenge the dichotomous view by highlighting the diversity and dynamics of living arrangement preferences in China and East Asia. Although coresidence remains a preferred arrangement for many (Logan & Bian, 1999; Sereny, 2011; Takagi & Silverstein, 2006; Zhang et al., 2014), younger generations and those with higher socioeconomic status show a declining preference for intergenerational coresidence (Sereny, 2011). Research into the alignment between preferred and actual living arrangements has revealed the dynamic nature of these changes (Gu et al., 2019; Xu et al., 2019). For instance, Sun and Zimmer (2022) found that although some older Chinese adults consistently live independently or coreside, many experience transitions between these arrangements. Moreover, less than half maintain concordance between preferred and actual living arrangements, with notable shifts in both directions. These patterns suggest that, even in societies with strong filial norms, living arrangement preferences are more dynamic than previously assumed.

### Grandparenting and preferences for coresidence

In East Asia in general and China in particular, research on preferred and actual living arrangements has largely centered on the influence of filial norms, emphasizing adult children’s obligations—particularly those of sons—to respect and support their parents through intergenerational coresidence. Although these norms remain influential, emerging studies highlight the growing importance of reciprocity in shaping intergenerational exchanges. For instance, parents who provide assistance with housework, childcare, or wedding expenses are more likely to receive financial support from their children (Brasher, 2022). Building on this perspective, our study shifts attention to how older adults perceive their responsibilities toward their adult children and how these perceptions influence their living arrangement preferences. Specifically, we examine grandparental childcare as a key dimension of intergenerational support, investigating whether and how caring for grandchildren shapes older adults’ preferences for coresidence. By exploring this reciprocity-driven dynamic, we contribute to a more nuanced understanding of how shifting familial expectations and obligations influence older adults’ residential choices in contemporary China.

Our analysis is grounded in the intergenerational solidarity theory (Bengtson & Roberts, 1991). This framework conceptualizes family relationships across generations as sustained through six dimensions: affectual, associational, consensual, functional, normative, and structural solidarity. These dimensions capture elements such as emotional closeness, interaction frequency, value alignment, exchanges of support, normative obligations, and spatial proximity. The theory posits that greater intergenerational solidarity strengthens cohesion and support between generations. Chen et al.’s (2011) application of intergenerational solidarity theory to China’s context suggests that grandparenting, as a form of functional solidarity, enhances coresidence (a form of structural solidarity).

Although early formulations of intergenerational solidarity theory primarily addressed family structure, later work distinguishes between latent solidarity (filial norms) and manifest solidarity (filial behaviors) (Silverstein et al., 2006). This distinction underscores that solidarity behaviors are often activated by situational needs. When intergenerational needs arise, latent norms manifest in supportive behaviors; when these needs decline, the behaviors tend to subside. Notably, unlike older adults’ long-term care needs, adult children’s need for childcare support is temporary, typically linked to a specific period in a child’s upbringing. This temporality allows for flexible living arrangements, with older adults transitioning between independent living and coresidence in response to grandchild care needs. Accordingly, living arrangement preferences may shift with the episodic need for grandparenting.

## Research hypotheses

This study seeks to understand older adults’ living arrangement preferences, particularly the choice between independent living and coresidence. We focus on how adult children’s needs for various forms of support—particularly grandparenting—relate to the older generation’s preferences for coresidence. In this study, we use the term “grandparenting” specifically to refer to older adults providing childcare support to their adult children. Although grandparenting can encompass a broader range of interactions and relationships, our focus is on its role in grandchild care and its implications for living arrangement preferences.

Drawing on intergenerational solidarity theory, we argue that when adult children express need for support—particularly in areas such as childrearing—older adults are more likely to prefer coresidence as a means of fulfilling these familial obligations. Living in close proximity offers several advantages over living at a distance, including more frequent interactions and stronger emotional support (Bao, 2022). We differentiate between adult children’s instrumental and emotional needs, predicting that both types will more strongly encourage preferences for coresidence than financial needs, which can be met without coresidence. Hence, we hypothesize:

*H1 (Effect of Adult Children's Needs): Adult children's instrumental and emotional needs are positively associated with older adults’ preference for intergenerational coresidence, while financial needs are not.*

Grandparenting is a unique form of intergenerational support that addresses both instrumental and emotional needs of adult children, alleviating work-related pressures and promoting intergenerational solidarity (Xu et al., 2017). Following intergenerational solidarity theory, we posit that the ethical dimension of solidarity gives a strong sense of purpose to grandparenting (functional solidarity) and a moral foundation to coresidence (structural solidarity). Thus, older adults who believe it is a parental duty to help raise the third generation are more inclined toward coresidence, while those who distance themselves from traditional beliefs in solidarity are less likely to prefer coresidence. In this study, we define “grandparenting beliefs” as individual attitudes and perceptions regarding grandparenting, particularly whether an older adult personally feels responsible for providing childcare. We propose:

*H2 (Effect of Grandparenting Beliefs): Older adults with stronger grandparenting norms are more likely to prefer intergenerational coresidence.*

We then consider grandparenting experiences and their direct influence on living arrangement preferences. Consistent with the ethical emphasis of intergenerational solidarity in China, we hypothesize that fulfilling grandparenting responsibilities reinforces ethical commitments toward the family and coresidential preferences. We therefore propose:

*H3 (Effect of Grandparenting Experiences): Older adults who have engaged in grandparenting are more likely to prefer intergenerational coresidence.*

The intensity of grandparents’ childcare is another factor that may shape coresidential preferences. In societies with weaker grandparenting norms, infrequent childcare often fosters positive attitudes toward grandparenting and coresidence, while intensive childcare may feel burdensome, reducing coresidential preferences (Fuller-Thomson & Minkler, 2001). It remains unclear if this pattern applies to China, where strong grandparenting norms shape intergenerational relationships (Chen et al., 2011). Drawing on intergenerational solidarity theory, we argue that intensive grandparenting in China is embedded within reciprocal exchanges that reinforce family cohesion. When older adults provide frequent childcare, they may experience stronger emotional bonds with their children and a heightened sense of familial duty, making coresidence a more attractive or even expected arrangement. Moreover, in urban settings like Shanghai, where logistical and economic conditions facilitate grandchild care, the potential burdens of intensive grandparenting may be mitigated. Thus, rather than deterring coresidential preferences, intensive grandparenting may enhance the desire for shared living.

*H4 (Effect of Grandparenting Intensity): Older adults who provide intensive grandparenting are more likely to prefer coresidence.*

Finally, we consider potential indirect pathways, such as grandparenting experiences reinforcing grandparenting beliefs, which in turn increase coresidential preferences. Although data limitations restrict our analysis of some pathways, we explore the possible effects of grandparenting experiences on grandparenting beliefs, and we propose:

*H5 (Indirect Effect of Grandparenting Experiences and Beliefs): Grandparenting experiences likely increase coresidential preferences indirectly through grandparenting beliefs and intensity, and grandparenting beliefs may increase coresidential preferences through grandparenting intensity.*

## Data and measurement

This study utilizes data from the 2022 Lifelong Education for Aging Productively in Shanghai survey (LEAP-SH). A stratified sampling design was implemented to cover 10 districts in Shanghai, with each district contributing two subdistricts (streets) or towns. From each of these areas, two residential or village committees were selected, totaling 40 committees. Within each committee, a quota sample of over 40 older adults was collected, balanced by gender and age group. All interviews were conducted with respondents’ informed consent, and missing data on variables such as marital status and pension income were handled using multiple imputation to enhance data completeness and reliability. After screening for eligibility, the final survey sample comprised 1,707 adults aged 55–85. Given this study’s focus on grandparenting, respondents without grandchildren were excluded, resulting in an analytical sample of 1,250 individuals.

The dependent variable in this study is the *preference for coresidence*, operationalized as a binary variable (1 = preference for living with children or grandchildren; 0 = preference for independent living). We also include actual residence in the descriptive (and supplementary) analysis, as the discrepancy between preferred and actual residence holds both theoretical importance and empirical relevance. It is a binary variable coded 1 if the interviewee lives with a child or grandchild, and 0 otherwise. Key independent variables include measures of adult children’s needs for parental support, grandparenting norms, grandparenting experiences, and grandparenting intensity. Several demographic and socioeconomic control variables are incorporated, such as gender, age, education level, pension income, homeownership, migrant status, self-reported health, and community engagement. These controls capture respondents’ available resources and demographic characteristics, allowing a more accurate assessment of the relationships among grandparenting, needs, and living arrangement preferences.

*Needs* are measured through a set of questions regarding the ways and frequency of the support respondents currently provide to their children. These questions cover six distinct forms of assistance, including *financial support, housework, grandparenting, companionship, problem-solving* (excluding household chores), *and home care*. Needs are operationalized through a series of binary variables representing different types of support that respondents provide to their adult children. *Financial support* is coded 1 if respondents report occasionally providing monetary assistance. *Housework* is coded 1 if respondents report occasionally assisting their children with housework. *Grandparenting* is coded 1 if respondents report occasionally providing care for their grandchildren. *Companionship* is coded 1 if respondents report occasionally offering companionship to their children. *Problem-solving* is coded 1 if respondents report occasionally helping their children with problem-solving (excluding household chores). *Home care* is coded 1 if respondents report occasionally providing caregiving when their children are sick. Each type of support is measured as a distinct binary variable to allow for detailed analysis of the forms of support potentially influencing coresidential preferences.

Grandparenting variables encompass norms, experiences, and intensity. *Grandparenting beliefs* are gauged through respondents’ level of agreement with the statement, “Grandparents should help care for grandchildren,” with responses categorized into two groups: agree and disagree. *Grandparenting experiences* are captured by whether respondents have ever provided care to their grandchildren (1 = yes, 0 = no). Although grandparenting norms and experiences could theoretically influence each other in a bidirectional manner, the data capture only past grandparenting experiences and current beliefs, restricting us to focusing on the potential influence of past grandchild care on current normative beliefs. Finally, *grandparenting intensity* is measured based on the frequency of childcare provided to each grandchild. Respondents were asked to report the frequency of their grandchild care, with four possible response options: daily, —one to six times per week, —one to three times per month, and once every few months. High-intensity grandchild care was defined and coded 1 under one of two conditions: when grandparents care for a single grandchild more than once per week, or when they care for two or more grandchildren simultaneously.

## Analytical strategies

We first presented the sample characteristics to provide an overview of the data. To test Hypothesis 1, we employed a logistic regression model to examine the association between adult children’s needs and older parents’ coresidence preferences. To further explore the relationship between grandparenting and coresidence preferences, we conducted a path analysis using generalized structural equation modeling (GSEM) in Stata 17.0. Path analysis is a robust statistical approach for investigating multiple pathways and sequential relationships among grandparenting beliefs, experiences, and intensity. Approximately 3%–4% of the data were missing for marital status and pension income, and these missing values were addressed using multiple imputation techniques. In the supplementary analysis, we further examined the relationship between grandparenting and actual living arrangements.

## Results

Table 1 provides a descriptive overview of these variables (the correlation matrix of all variables is presented in the Supplementary Table A1). In terms of actual living arrangements, around 25% of older residents live with their children, the majority—nearly 75%—now reside either alone or only with a spouse. Notably, an even higher proportion—approximately 90%—expresses a preference for independent living. Nonetheless, nearly 50% report having engaged in grandparenting. In terms of grandparenting beliefs, 54% of respondents agree with the view that grandparents should assist in grandchild care, while 46% disagree.

**Table 1.** Descriptive analysis of dependent, independent, and control variables.

Variable	%/Mean	Variable	%/Mean
Coresidence preferences		Gender	
No (ref.)	89.52	Male (ref.)	38.72
Yes	10.48	Female	61.28
Actual coresidence		Age	
No (ref.)	74.40	55–64 (ref.)	21.20
Yes	25.60	65–74	55.36
Grandparenting beliefs		75+	23.44
Negative attitude (ref.)	45.76	Marital status	
Positive attitude	54.24	Single/divorced/widow/otherwise (ref.)	17.84
Grandparenting intensity		Married/cohabiting	82.16
Low intensity(ref.)	66.56	Migrant status	
High intensity	33.44	Local resident (ref.)	83.84
Previous grandparenting experiences		Migrant	16.16
No (ref.)	49.28	Education	
Yes	50.72	Elementary school and below (ref.)	19.92
Financial support needs		Secondary school	67.68
No (ref.)	71.20	Postsecondary school	12.40
Yes	28.80	Log means of pension income (*SD*)	4.60 (0.44)
Housework support needs		Homeowner	
No (ref.)	52.96	No (ref.)	18.24
Yes	47.04	Yes	81.76
Grandparenting needs		Self-report health	
No (ref.)	36.88	Poor (ref.)	15.68
Yes	63.12	Fair	42.16
Companionship needs		Good	42.16
No (ref.)	32.88	Community engagement	
Yes	67.12	Negative (ref.)	23.20
Problem-solving needs		Neutral	15.36
No (ref.)	40.48	Positive	61.44
Yes	59.52	*N*	1,250
Home care needs			
No (ref.)	61.68		
Yes	38.32		

Data source: 2022 Lifelong Education for Aging Productively in Shanghai (LEAP-SH).

These descriptive patterns highlight a nuanced dynamic between independent living preferences and the substantial engagement in and normative support for grandparenting. This paradox suggests that although older adults in Shanghai may value their independence, many still adhere to and actively participate in grandparenting responsibilities. In the subsequent analyses, we examine how these seemingly contradictory preferences are managed.

Table 2 presents the results testing Hypothesis 1, which posits that adult children’s instrumental and emotional needs will be associated with a greater preference for intergenerational coresidence, while financial needs will not. The findings in Table 2 lend strong support to this hypothesis. Parental support with household work, companionship, childcare, and problem-solving is associated with greater odds of preferring coresidence. Consistent with expectations, financial needs are not linked to coresidential preferences, suggesting that monetary support alone does not necessitate shared living. Notably, the effect sizes are more pronounced for housework assistance and companionship compared to grandparenting and problem-solving support. Interestingly, the need for home care is not significantly associated with coresidential preference, potentially because this type of care is less frequent.

**Table 2.** Logistic regression analysis of parental support and coresidence preferences (odds ratio).

Variable	Model 1	Model 2	Model 3	Model 4	Model 5	Model 6
Financial support needs	1.23					
Housework support needs		3.25***				
Grandparenting needs			1.96**			
Companionship needs				3.13***		
Problem-solving needs					1.44+	
Home care needs						0.87
Woman	1.46+	1.40	1.39	1.43	1.46+	1.44
Age						
65–74	1.09	1.22	1.12	1.09	1.10	1.06
75+	0.73	0.94	0.86	0.83	0.76	0.70
Married/cohabiting	0.49**	0.44***	0.45***	0.47***	0.48***	0.49**
Migrant	1.22	0.93	1.08	1.09	1.19	1.21
Education						
Secondary school	0.46**	0.47**	0.46**	0.47**	0.46**	0.46**
Postsecondary School	0.37*	0.38*	0.36*	0.37*	0.36*	0.36*
Log mean of pension income	1.26	1.30	1.25	1.34	1.27	1.26
Homeowner	0.61*	0.59*	0.59*	0.60*	0.62*	0.60*
Self-report health						
Fair	0.73	0.74	0.72	0.71	0.74	0.74
Good	1.42	1.37	1.35	1.30	1.40	1.43
Community engagement						
Neutral	1.09	1.10	1.10	1.08	1.07	1.11
Positive	0.87	0.83	0.87	0.84	0.84	0.89
Observations	1,250	1,250	1,250	1,250	1,250	1,250
McFadden’s *R*²	0.062	0.100	0.072	0.087	0.064	0.061
Log likelihood	−393.493	−377.571	−389.309	−382.774	−392.357	−393.750

*Note*. Data source: 2022 Lifelong Education for Aging Productively in Shanghai (LEAP-SH). Odds ratios in the table are obtained from logistic regression models.

*** *p* < .001.

** *p* < .01.

* *p* < .05.

+ *p* < .1.

Additional findings merit attention. First, having a spouse significantly reduces the likelihood of preferring coresidence, which helps explain findings from China’s 2020 population census: living with a spouse is the most common arrangement for older adults (43.7%), while only 23.1% live with both spouse and children (Zhao, 2023). Second, educational attainment greatly diminishes the preference for coresidence. Those with a secondary education are approximately half as likely as those with primary or no education to prefer coresidence, and those with postsecondary education are even less likely. Rather than facilitating coresidence, education appears to shift preferences toward modern, independent living ideals. Third, homeownership is associated with a reduced preference for coresidence, consistent with research suggesting that shared living often reflects financial necessity. Finally, age and health status, which are critical determinants of actual living arrangements, do not significantly impact preferences, highlighting a conceptual distinction between preferred and actual living arrangements.

**Figure 1.**
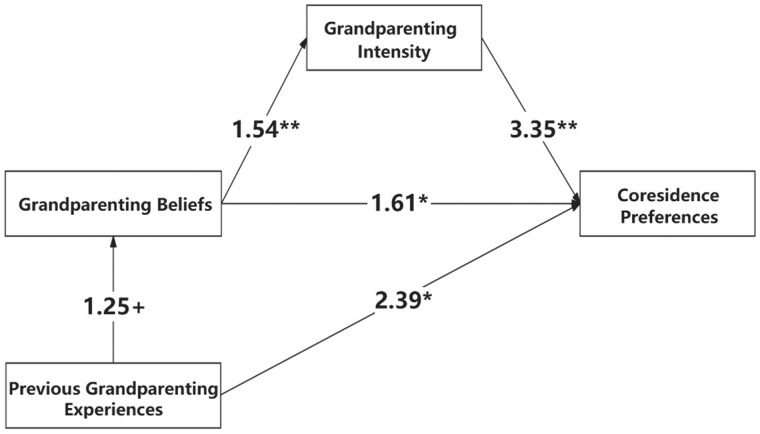
Path analysis of the effects of grandparenting beliefs, previous experiences, and intensity on coresidence preferences (odds ratio). Data source: 2022 Lifelong Education for Aging Productively in Shanghai (LEAP-SH). Odds ratios in the figure are obtained from the generalized structural equation modeling (GSEM) models. Models controlled age, gender, migration status, marital status, education, pension income, homeownership, self-reported health, and community engagement. **p < .01. *p < .05. + *p* < .1.

Following the evidence in Table 2 that grandparenting needs influence coresidential preferences, we further examine grandparenting beliefs, previous experiences, and intensity as pathways shaping these preferences. Results from generalized structural equation modeling are presented in Table 3, with a graphical summary in Figure 1.

**Table 3.** The GSEM modeling of grandparenting beliefs, intensity, previous experiences, and coresidence preferences (odds ratio).

Variable	Coresidence preferences	Grandparenting intensity	Grandparenting beliefs
Grandparenting beliefs	1.61*	1.54**	.
Grandparenting intensity	3.35**	.	
Previous grandparenting experiences	2.39*		1.25+
Female	1.37	1.12	0.93
Age			
65–74	1.10	0.74+	1.00
75+	0.87	0.16***	0.70+
Married/cohabiting	0.44***	1.62*	1.10
Migrant	1.10	2.07***	1.14
Education			
Secondary school	0.46**	1.89**	0.59**
Postsecondary school	0.36*	3.05***	0.51**
Log mean of pension income	1.31	0.97	0.73+
Homeowner	0.62*	1.04	0.86
Self-report health			
Fair	0.76	1.02	0.83
Good	1.40	1.14	1.04
Community engagement			
Neutral	1.13	0.81	1.01
Positive	0.89	0.83	0.93
Observations	1,250	1,250	1,250

*Note*. GSEM = generalized structural equation modeling. Data source: 2022 Lifelong Education for Aging Productively in Shanghai (LEAP-SH). Odds ratios in the table are obtained from logistic regression models.

*** *p* < .001.

** *p* < .01.

* *p* < .05.

+ *p* < .1.

Hypothesis 2 predicts a direct influence of grandparenting norms on coresidential preferences. Figure 1 shows that, relative to those who disagree with the statement that grandparents should assist in caring for grandchildren, those who agree are 60% more likely to prefer coresidence. These findings support Hypothesis 2, indicating that stronger grandparenting beliefs are associated with a higher likelihood of preferring coresidence.

Hypothesis 3 posits a direct effect of grandparenting experiences on coresidential preferences. Figure 1 shows that respondents with grandparenting experience are twice as likely to prefer coresidence compared to those without, affirming Hypothesis 3.

Hypothesis 4 explores the effect of grandparenting intensity on living preferences, an area with limited prior research. Evidence from Figure 1 indicates that high-intensity caregivers are more than twice as likely to prefer coresidence as those who provide occasional or no care, supporting Hypothesis 4.

Hypothesis 5 examines indirect pathways connecting grandparenting experiences and norms to coresidential preferences. Figure 1 demonstrates that, beyond the direct effect, grandparenting experiences indirectly increase coresidential preference through the mediation of grandparenting beliefs. Respondents with grandchild care experience are 25% more likely to endorse the norm that grandparents should assist in childcare. Likewise, a portion of the influence of grandparenting beliefs on coresidential preferences is mediated by grandparenting intensity. Specifically, those who agree with the norm are 1.54 times as likely to engage in high-intensity grandchild care as those who disagree. These findings provide strong support for Hypothesis 5.

Although the relationship between grandparenting and actual living arrangements is not the primary focus of this study, our supplementary analyses (Table A2 to Table A4) reveal that grandparenting beliefs, the intensity of grandchild care, and past grandparenting experiences are all positively associated with the likelihood of actual coresidence.

In summary, grandparenting factors—beliefs, experiences, and intensity—are all positively associated with coresidential preferences. These factors are also interconnected: grandparenting experiences bolster grandparenting beliefs, which then encourage high-intensity grandchild care. Consequently, grandparenting experiences not only directly impact coresidential preferences but also exert indirect effects via beliefs and grandchild care intensity. Similarly, the influence of grandparenting beliefs on coresidential preference is partially mediated by grandchild care intensity, supporting the theory of intergenerational solidarity and offering substantial empirical evidence from Shanghai.

Several demographic and socioeconomic factors show either direct or indirect effects on coresidential preferences. Age differences are insignificant for grandparenting beliefs and preferences. However, they are significantly associated with the intensity of grandparenting involvement. As parents age, they are less likely to engage in high-intensity grandchild care activities. Having a spouse lowers coresidential preference but raises the likelihood of high-intensity grandchild care, which in turn elevates coresidential preference. Compared to local residents, migrant older adults are more likely to participate in high-intensity grandparental grandchild care. Higher educational attainment reduces both coresidential preference and adherence to grandparenting norms, though educated respondents who participate in grandchild care tend to do so intensely. Homeownership reduces coresidential preference but does not affect grandparenting norms or intensity. Pension income, self-reported health, and community engagement do not significantly impact grandparenting norms, grandchild care intensity, or living preferences.

## Conclusion and discussion

Using representative data from 2022 in Shanghai, this study provides an updated analysis of the living arrangement preferences of older adults, with a particular focus on the role of grandparenting. We find that adult children’s instrumental and emotional needs are positively associated with older adults’ preference for intergenerational coresidence, whereas financial needs show no significant association. Furthermore, older adults who hold stronger grandparenting beliefs and have prior grandparenting experiences are more likely to prefer intergenerational coresidence. Interestingly, those who provide high-intensity grandparenting exhibit an even stronger preference for coresidence. We also examine the relationship between grandparenting and actual coresidence, while controlling for living arrangement preferences. Notably, we find a positive association between all three dimensions of grandparenting—beliefs, intensity, and past experiences—and the likelihood of coresidence (see Supplementary Table 1). This suggests that some older adults opt to coreside with their children due to grandparenting responsibilities, even though they express a preference for independent living.

Our study highlights a profound shift in living arrangements in China, particularly in Shanghai, where a decisive move toward independent living is evident. The majority of Shanghai residents—nearly 75%—now live alone or only with a spouse, while 90% express a preference for independent living. This finding is striking given China’s deep-rooted filial norms, which have traditionally emphasized intergenerational coresidence. Nationally, China’s 2020 population census data reflect a similar pattern, with one-generation households emerging as the most common arrangement—rising from 21.7% in 2000 to 34.2% in 2010, and reaching 49.5% in 2020. Among adults aged 60 and older, 58.4% of men and 52.7% of women either lived alone or with a spouse in 2020 (Zhao, 2023). Consistent with these national trends, our analysis underscores a fundamental shift toward independent living, whether in preferences or actual arrangements.

The strong preference for independent living raises an essential question: how do families reconcile this desire for independence with the intergenerational support often facilitated through coresidence? Although prior studies have largely focused on adult children providing care to aging parents, our research shifts the lens to older adults’ responses to their children’s needs, particularly in relation to childcare. We find that older adults adapt their living arrangement preferences in response to their adult children’s practical and emotional needs, such as assistance with housework, childcare, companionship, and problem-solving. Interestingly, financial needs do not typically prompt a preference for coresidence, as these can typically be met without shared living. This highlights the flexibility and situational nature of living arrangement preferences.

Previous research suggests that actual living arrangements in China are adapting to shifting demographic trends and evolving family dynamics. As spousal caregiving becomes more prominent, coresidence as the primary source of care for aging parents has declined. When a spouse is unavailable or unable to fulfill the caregiving role, adult children are expected to step in, often through coresidence (Gruijters, 2017). Parental age, health, and widowhood prompt living arrangement adjustments, with functional limitations particularly influencing the decision to coreside (Zimmer, 2005). Our findings extend this understanding, showing that not only do adult children adjust their actual living arrangements to accommodate parental needs (as found in previous literature, such as Chen, 2005; Wen & Gu, 2021; Zhang et al., 2014; Zimmer, 2005), but parents also adapt their living arrangement preferences in response to the needs of their adult children.

The literature on intergenerational relationships from the perspective of grandparenting has established a link between caring for grandchildren and intergenerational coresidence. Chen et al. (2011) argue that, unlike in societies where grandparenting norms are ambiguous and grandparents have limited involvement in childcare, grandparenting is more prevalent in China, contributing to a comparatively higher level of intergenerational coresidence. A less explored question is whether this shift to coresidence is a voluntary choice or driven by societal norms and expectations. Our analysis reveals a clear connection between grandparenting and preferences for coresidence, suggesting that grandparents in Shanghai largely embrace this role willingly and actively. Notably, although independent living is preferred in general, preferences shift toward coresidence when grandparenting is involved. This finding underscores the adaptability of family structures in response to grandchild care responsibilities.

Moreover, our study advances the understanding of how grandparenting shapes preferences for coresidence by uncovering the mechanisms behind this link. Both directly and indirectly, involvement in grandparenting reinforces a preference for coresidence. The grandparenting belief—whereby grandparents are expected to assist with childcare—is strongly associated with a higher preference for living together. Although prior research has documented the health burdens of intensive grandchild care on older adults (Liu & Chen, 2022; Song et al., 2022), the impact of high-intensity grandparenting on preferences for intergenerational coresidence remains understudied. Addressing this gap, our analysis provides direct evidence that, in the highly urbanized context of Shanghai, intensive grandparenting is positively linked to a stronger preference for coresidence.

Our findings offer a contemporary perspective that revises the pattern identified by Logan and Bian (1999). Their research suggested that intergenerational coresidence in urban China weakened filial norms, a key factor influencing preferences for shared living. However, the landscape has changed since the 1990s, when the potential strains of shared living and grandchild care may have eroded filial expectations. In the 2020s, improved housing, modern appliances, and enhanced community services have likely alleviated many of these stressors. As a result, the relationship between high-intensity grandparenting and preferences for intergenerational coresidence in contemporary urban China appears to have evolved, reflecting shifting social and material conditions. It is also possible that our measure of high-intensity grandparenting—based on the frequency of childcare—does not fully capture the variation in care intensity or the associated role strain and family stress. This represents a limitation that future research should aim to address.

Overall, our study contributes to the literature on intergenerational relationships and living arrangements by highlighting the adaptability of older adults in China as they navigate shifting demographics and family structures. Although the majority are able to pursue their preference for independent living, those engaged in grandparenting adjust their preferences accordingly. This malleability mirrors adjustments in actual living arrangements to accommodate the needs of both aging parents and adult children. Our findings align with patterns observed from other East Asian contexts, where intergenerational family life is characterized by “intermittent but intensive forms of support that punctuate periods of relative autonomy between generations” (Silverstein et al., 2006).

Our findings from Shanghai support the intergenerational solidarity theory, particularly emphasizing normative solidarity as a key force shaping Chinese family dynamics. This normative dimension influences other forms of intergenerational support, such as functional solidarity (evident in grandparenting) and structural solidarity (manifest in coresidence). Chinese scholars have long examined the ethical foundation of intergenerational relationships. Fei (1983) described a long-standing “feedback model” where the first generation nurtures the second, supports the third, and expects reciprocation in old age (Yi, 1986). Yang and He (2004) extended this model to contemporary China, noting that rising living standards and improved longevity delay the point at which parents require substantial help from their children. This generational shift has reinforced the “ethical responsibility” among older parents, encouraging self-reliance to ease their children’s burdens. Yan’s (2016, 2018) concept of descending familism, or neo-familism, further underscores the role of ethical obligations in modern Chinese family life. This model highlights the intergenerational sacrifice of personal interests to serve the family unit, with a life focus oriented toward grandchildren. Scholars have also noted the strong affectual bond between grandparents and grandchildren, which contributes significantly to the psychological well-being of both generations (Liu, 2017; Xu et al., 2014). Our analysis aligns with these perspectives, suggesting that older adults who hold strong normative beliefs about grandparenting are more likely to prefer coresidence and to provide childcare, even when such involvement may entail intensive childcare responsibilities.

Our research also raises important questions for future studies. Although previous research has established that age and health significantly influence actual living arrangements (Chen, 2005; Zhang et al., 2014; Zimmer, 2005), we find that these factors do not significantly affect older adults’ preferences for coresidence. This discrepancy underscores the need to conceptually and empirically distinguish between preferred and actual living arrangements. A key avenue for future research is to examine whether this misalignment contributes to living arrangement discordance. In connection, further investigation is needed to understand the factors driving the convergence of preferences for independent living across age groups and health statuses. One possibility is that older adults’ preference for independent living—even in the face of declining health—stems from a desire to reduce the burden on their adult children. Although our data do not directly measure this motivation, the pattern of findings—particularly the emphasis on normative obligations and active grandparenting involvement—may be consistent with an underlying desire to reduce burdens on adult children. The role of this potential motivation in shaping living arrangement preferences should be a fruitful area for future research.

Another critical question for future research is whether the ethical foundation of grandparenting norms is gradually weakening. Our findings indicate that 45% of older adults with grandchildren in Shanghai no longer view grandparental childcare as an obligation. If this shift continues, adherence to traditional grandparenting expectations may decline, potentially reshaping both preferred and actual living arrangements as well as broader intergenerational relationships. Although this study does not directly address these dynamics, future research would benefit from systematically comparing older adults who reject grandparenting obligations with those who maintain strong grandparenting beliefs. Key areas of investigation include differences in living arrangement preferences, the extent of living arrangement concordance, and the potential implications for their psychological well-being. Such analyses could provide deeper insights into how evolving attitudes toward grandparenting influence intergenerational support patterns and family cohesion in aging societies.

Another avenue for future research concerns the implications of China’s planned increase in the retirement age, set to begin in 2025. Currently, the relatively low retirement age—50 for women in blue-collar jobs, 55 for women in white-collar jobs, and 60 for men—enables many middle-aged and older adults to actively participate in grandparenting. By 2040, however, the retirement age will gradually rise to 55 for blue-collar women, 58 for white-collar women, and 63 for men. This policy change raises critical questions about the potential role strain for affected older adults, who may need to navigate competing demands between work and grandchild care. Some may opt for early retirement to fulfill grandparenting responsibilities, while others may forgo or reduce grandchild care duties to extend their careers. Still, others may attempt to balance both roles, potentially leading to stress and exhaustion. Future research should explore how these changes affect grandparenting norms and beliefs, particularly whether they lead to a decline in grandparental involvement or a restructuring of grandchild care arrangements within families. Additionally, examining how variations in socioeconomic status, occupational flexibility, and family expectations shape older adults’ responses to the new retirement policies would offer valuable insights into the evolving intergenerational landscape in China.

We acknowledge several limitations in our current study. First, our analysis is based on cross-sectional data, which captures a snapshot in time but does not allow us to examine changes in grandparenting beliefs and behaviors over time. As social norms, economic conditions, and policies evolve, older adults’ preferences and practices may shift in ways that cannot be fully captured in a single wave of data. Future research should employ longitudinal designs to track these dynamics, shedding light on how grandparenting expectations, living arrangement preferences, and intergenerational relationships evolve across different life stages and policy changes. Longitudinal studies would also allow for a deeper understanding of causal relationships, such as whether changing labor market participation influences grandparenting norms, beliefs, and behaviors, preferred and actual living arrangements, and patterns of intergenerational relationships.

Second, our study focuses exclusively on Shanghai, a highly developed and economically vibrant metropolis with unique demographic, social, and policy contexts. Although Shanghai provides an important case for studying grandparenting and living arrangements in urban China, its findings may not fully generalize to other regions, particularly smaller cities, rural areas, or provinces with different cultural and economic conditions. Variations in local pension systems, housing conditions, and migration patterns could shape older adults’ grandchild care roles and living arrangements in ways that differ from those observed in Shanghai. Alternatively, sweeping societal changes may be occurring nationwide, diminishing regional differences and making Shanghai’s experience more representative of broader trends. In this context, findings from Shanghai often serve as leading indicators for shifts that may eventually unfold in other urban areas across China. However, which scenarios hold better remains an open question. Future research should extend this line of inquiry to other parts of China, enabling comparative analyses that can clarify how regional differences influence grandparenting beliefs and intergenerational support.

## Supplementary material

Supplementary material is available at *The Journals of Gerontology, Series B: Psychological Sciences and Social ­Sciences* online.

## Supplementary Material

gbaf138_Supplementary_Data

## Data Availability

The data are available upon request. This study was not preregistered.
