# Supplementary material for: Grandparenting and coresidence preferences in Shanghai, China: the influence of grandparenting beliefs, experiences, and intensity
Source: J Gerontol B Psychol Sci Soc Sci. 2025 Jul 25;80(10):gbaf138. doi: 10.1093/geronb/gbaf138 (PMC12462770; doi:10.1093/geronb/gbaf138)
Supplement: gbaf138_Supplementary_Data [file gbaf138_supplementary_data.zip › gbaf138_Supplementary_Tables.docx]

Supplementary Tables

Table A1. The correlation matrix of all variables

|  | Coresidence preferences | Actual coresidence | Beliefs | Intensity | Experiences | Female | Age | Married | Migrant | Education | Pension | Homeowner | Health | Community |
| --- | --- | --- | --- | --- | --- | --- | --- | --- | --- | --- | --- | --- | --- | --- |
| Coresidence preferences | 1.000 |  |  |  |  |  |  |  |  |  |  |  |  |  |
| Actual coresidence | 0.374 | 1.000 |  |  |  |  |  |  |  |  |  |  |  |  |
| Beliefs | 0.105 | 0.167 | 1.000 |  |  |  |  |  |  |  |  |  |  |  |
| Intensity | 0.062 | 0.284 | 0.096 | 1.000 |  |  |  |  |  |  |  |  |  |  |
| Experiences | 0.003 | -0.155 | 0.045 | -0.719 | 1.000 |  |  |  |  |  |  |  |  |  |
| Female | 0.074 | 0.049 | 0.018 | -0.008 | 0.061 | 1.000 |  |  |  |  |  |  |  |  |
| Age | -0.028 | -0.090 | -0.068 | -0.274 | 0.191 | -0.060 | 1.000 |  |  |  |  |  |  |  |
| Married | -0.107 | -0.038 | 0.017 | 0.113 | -0.025 | -0.156 | -0.172 | 1.000 |  |  |  |  |  |  |
| Migrant | 0.034 | 0.236 | 0.046 | 0.177 | -0.150 | -0.066 | -0.145 | 0.017 | 1.000 |  |  |  |  |  |
| Education | -0.117 | -0.091 | -0.131 | 0.116 | -0.100 | -0.226 | -0.003 | 0.064 | 0.070 | 1.000 |  |  |  |  |
| Pension | -0.059 | -0.205 | -0.124 | -0.022 | -0.016 | -0.230 | 0.100 | 0.057 | -0.209 | 0.407 | 1.000 |  |  |  |
| Homeowner | -0.082 | -0.145 | -0.051 | -0.047 | 0.036 | -0.014 | 0.026 | 0.040 | -0.322 | 0.044 | 0.165 | 1.000 |  |  |
| Health | 0.045 | 0.047 | 0.021 | 0.044 | -0.020 | -0.094 | -0.043 | 0.082 | 0.017 | 0.042 | 0.018 | -0.011 | 1.000 |  |
| Community | -0.041 | -0.016 | -0.050 | 0.000 | 0.005 | 0.094 | -0.022 | 0.036 | -0.040 | 0.214 | 0.148 | 0.107 | 0.121 | 1.000 |

Data source: 2022 LEAP-SH.

Table A2. Logistic Regression Models of Actual Coresidence with Adult Children Based on Grandparenting Dimensions and Control Variables

|  | Model 1 | Model 2 | Model 3 | Model 4 |
| --- | --- | --- | --- | --- |
| Grandparenting beliefs | 1.85*** | 1.71*** | 1.93*** | 1.65** |
| Grandparenting intensity |  | 3.98*** |  | 6.27*** |
| Previous grandparenting experiences |  |  | 0.44*** | 1.75+ |
| Coresidence preference | 11.33*** | 11.87*** | 12.16*** | 11.48*** |
| Female | 0.94 | 0.90 | 0.98 | 0.87 |
| Age |  |  |  |  |
| 65-74 | 0.64* | 0.68* | 0.68* | 0.67* |
| 75+ | 0.60* | 0.93 | 0.74 | 0.91 |
| Married/cohabiting | 0.94 | 0.82 | 0.96 | 0.78 |
| Migrant | 3.10*** | 2.61*** | 2.74*** | 2.67*** |
| Education |  |  |  |  |
| Secondary school | 0.97 | 0.80 | 0.93 | 0.79 |
| Post-secondary school | 0.83 | 0.59 | 0.74 | 0.59 |
| Log mean of pension income | 0.52*** | 0.49*** | 0.49*** | 0.50*** |
| Houseowner | 0.79 | 0.76 | 0.78 | 0.76 |
| Self-report health |  |  |  |  |
| Fair | 1.06 | 1.05 | 1.04 | 1.06 |
| Good | 1.15 | 1.11 | 1.12 | 1.12 |
| Community engagement |  |  |  |  |
| Neutral | 0.84 | 0.94 | 0.87 | 0.95 |
| Positive | 1.20 | 1.33 | 1.24 | 1.33 |
| Observations | 1,250 | 1,250 | 1,250 | 1,250 |
| R^2^ | 0.190 | 0.240 | 0.209 | 0.243 |
| Log likelihood | -575.832 | -540.180 | -562.440 | -538.172 |

Notes: (1) Odds ratios in the table are obtained from logistic regression models.

(2) ***p<0.001; **p<0.01; *p<0.05, +p<0.1

Data source: 2022 LEAP-SH.

Table A3. The GSEM Modelling of Grandparenting Beliefs, Intensity (0-20 score) and Past Experiences on Coresidence Preferences (odds ratio)

|  | Coresidence Preferences | Grandparenting Intensity | Grandparenting Norms |
| --- | --- | --- | --- |
| Grandparenting beliefs | 1.69* | 2.04*** | . |
| Grandparenting intensity | 1.07* | . |  |
| Previous grandparenting experiences | 0.87 | 3.64*** | 1.25+ |
| Female | 1.40 | 1.35* | 0.93 |
| Age |  |  |  |
| 65-74 | 1.08 | 1.05 | 1.00 |
| 75+ | 0.73 | 1.10 | 0.70+ |
| Married/cohabiting | 0.46*** | 1.45* | 1.10 |
| Migrant | 1.08 | 1.93*** | 1.14 |
| Education |  |  |  |
| Secondary school | 0.51** | 0.89 | 0.59** |
| Post-secondary school | 0.40* | 1.03 | 0.51** |
| Log mean of pension income | 1.34 | 0.82 | 0.73+ |
| Houseowner | 0.63+ | 0.83 | 0.86 |
| Self-report health |  |  |  |
| Fair | 0.77 | 0.86 | 0.83 |
| Good | 1.42 | 1.04 | 1.04 |
| Community engagement |  |  |  |
| Neutral | 1.10 | 1.09 | 1.01 |
| Positive | 0.89 | 0.86 | 0.93 |
| Observations | 1,250 | 1,250 | 1,250 |

Notes: (1) Odds ratios in the table are obtained from logistic regression models.

(2) ***p<0.001; **p<0.01; *p<0.05, +p<0.1

Data source: 2022 LEAP-SH.

Table A4. The GSEM Modelling of Grandparenting Beliefs, Intensity and Past Experiences on Coresidence Preferences with Gender Interaction Terms (odds ratio)

|  | Coresidence Preferences | Grandparenting Intensity | Grandparenting Beliefs |
| --- | --- | --- | --- |
| Grandparenting beliefs | 1.90+ | 2.45** | . |
| Grandparenting intensity | 3.98* | . |  |
| Previous grandparenting experience | 2.10 | 0.00 | 1.20 |
| Beliefs*female | 0.79 | 1.74 |  |
| Intensity*female | 0.76 |  |  |
| Experience*female | 1.18 | 0.65 | 1.08 |
| Female | 1.67 | 1.27 | 0.90 |
| Age |  |  |  |
| 65-74 | 1.09 | 0.92 | 1.00 |
| 75+ | 0.87 | 0.22*** | 0.70+ |
| Married/cohabiting | 0.44*** | 2.93*** | 1.10 |
| Migrant | 1.11 | 1.36 | 1.15 |
| Education |  |  |  |
| Secondary school | 0.46** | 2.37** | 0.59** |
| Post-secondary school | 0.36* | 3.17** | 0.51** |
| Log means of pension income | 1.30 | 0.75 | 0.73+ |
| Houseowner | 0.62* | 1.10 | 0.86 |
| Self-report health |  |  |  |
| Fair | 0.76 | 1.05 | 0.83 |
| Good | 1.43 | 1.30 | 1.04 |
| Community engagement |  |  |  |
| Neutral | 1.13 | 0.72 | 1.01 |
| Positive | 0.89 | 0.77 | 0.93 |
| Observations | 1,250 | 1,250 | 1,250 |

Notes: (1) Odds ratios in the table are obtained from logistic regression models.

(2) ***p<0.001; **p<0.01; *p<0.05, +p<0.1

Data source: 2022 LEAP-SH.
